# ﻿On two new *Oedignatha* species from Xishuangbanna, China, and the first description of the female of *Jacaenamenglaensis* Mu & Zhang, 2020 (Araneae, Liocranidae)

**DOI:** 10.3897/zookeys.1144.97073

**Published:** 2023-02-02

**Authors:** Ying Lu, Chang Chu, Shuqiang Li, Zhiyuan Yao

**Affiliations:** 1 College of Life Science, Shenyang Normal University, Shenyang 110034, Liaoning, China Shenyang Normal University Shenyang China; 2 Institute of Zoology, Chinese Academy of Sciences, Beijing 100101, China Institute of Zoology, Chinese Academy of Sciences Beijing China

**Keywords:** Biodiversity, morphology, spiders, taxonomy, tropics

## Abstract

Liocranid spiders from the Xishuangbanna Tropical Botanical Garden in Yunnan, China are studied. Two new species of *Oedignatha* Thorell, 1881, *O.dian* Lu & Li, **sp. nov.** (♂♀) and *O.menglun* Lu & Li, **sp. nov.** (♂♀), are described, and the female of *Jacaenamenglaensis* Mu & Zhang, 2020 is described for the first time. The specimens studied are deposited in the Institute of Zoology, Chinese Academy of Sciences (IZCAS) in Beijing, China.

## ﻿Introduction

The spider family Liocranidae Simon, 1897 includes 35 extant genera and 312 extant species worldwide, of which 36 species of eight genera have been recorded from China (WSC 2022). They are wandering spiders, usually inhabiting forest litter or cultivated land. One of these genera, *Oedignatha* Thorell, 1881, was established based on a female spider, *Oedignathascrobiculata* Thorell, 1881, collected from India (Thorell 1881). Thorell’s (1881) description lacks figures, and Biswas (1984) published drawings of the female of *O.scrobiculata*. Thirty-nine *Oedignatha* species are known from South and Southeast Asia (WSC 2022). The type species of *Jacaena* Thorell, 1897, *J.distincta* Thorell, 1897, from Myanmar, also had no figures when it was originally described (Thorell 1897), and Deeleman-Reinhold (2001) later provided drawings of the type species. Since then, 15 new species of *Jacaena* have been reported, including three species transferred from *Sesieutes* Simon, 1897. The genus *Jacaena* is endemic to southeast Asia and currently contains 16 species (WSC 2022).

The Xishuangbanna Tropical Botanical Garden (XTBG) is managed by Chinese Academy of Sciences and is considered one of the most significant tropical rainforest nature reserves in Xishuangbanna, in southwestern Yunnan, China. The 1125-ha area of XTBG includes a 250-ha patch of well-preserved primary and secondary tropical rainforests, as well as some planted forests (e.g. rubber plantation and rubber–tea mixtures) (Zheng et al. 2015, 2017; Yao et al. 2018). From 2006 to 2020, more than 800 spider species have been reported from this 1125-ha garden (Li 2021; Yao and Li 2021).

Prior to the current study, two genera and five species of liocranid spiders have been described from Xishuangbana: *Jacaenaaspera* Mu & Zhang, 2020 (♂♀), *J.bannaensis* Mu & Zhang, 2020 (♂♀), *J.menglaensis* (♂), *J.zhui* (Zhang & Fu, 2011) (♂♀), and *Paratussinensis* Marusik, Zheng & Li, 2008 (♂♀). In this paper, we provide the first female description of *J.menglaensis* and describe two new species of *Oedignatha* discovered within the XTBG.

## ﻿Materials and methods

Specimens were examined and measured with a Leica M205 C stereomicroscope. Left male pedipalps were photographed and drawn. Epigynes were photographed before dissection. Vulvae were treated in a 10% warm solution of potassium hydroxide (KOH) to dissolve soft tissues before illustration. Images were captured with a Canon EOS 750D wide zoom digital camera (24.2 megapixels) mounted on the stereomicroscope mentioned above and assembled using Helicon Focus v. 3.10.3 image stacking software (Khmelik et al. 2005). All measurements are given in millimetres (mm). The median ocular area (MOA) refers to the area between the anterior median eyes and the posterior median eyes. Leg measurements are shown as: total length (femur, patella, tibia, metatarsus, tarsus). Leg segments were measured on their dorsal side. The species distribution map was generated with ArcGIS v. 10.2 (ESRI, Inc.). References to figures of cited papers are listed in lowercase (fig./figs); figures from this paper are noted with a capital letter (Fig./Figs). The specimens studied are preserved in 75% ethanol and deposited in the Institute of Zoology, Chinese Academy of Sciences (**IZCAS**) in Beijing, China.

Terminology and taxonomic descriptions follow Deeleman-Reinhold (2001) and Mu and Zhang (2020).

The following abbreviations are used in the descriptions:

**ALE** anterior lateral eye;

**AME** anterior median eye;

**d** dorsal;

**MOA** median ocular area;

**pl** prolateral;

**PLE** posterior lateral eye;

**PME** posterior median eye;

**pv** prolateral ventral;

**rl** retrolateral;

**rv** retrolateral ventral.

## ﻿Taxonomy

### ﻿Family Liocranidae Simon, 1897

#### ﻿Genus Jacaena

Taxon classificationAnimaliaAraneaeLiocranidae

Thorell, 1897

4EBA0964-5159-57C8-9C97-48FAEBA0F6A4

##### Type species.

*Jacaenadistincta* Thorell, 1897 from Myanmar.

##### Comments.

The genus is endemic to southeast Asia, and currently contains 16 species. Among these species, seven are known to occur in China: *J.aspera* (♂♀), *J.bannaensis* (♂♀), *J.jinxini* Liu & Xu, 2020 (♂♀), *J.luteolus* Mu & Zhang, 2020 (♂), *J.menglaensis* (♂), *J.tengchongensis* Zhao & Peng, 2013 (♀), and *J.zhui* (♂♀).

#### ﻿ Jacaena menglaensis

Taxon classificationAnimaliaAraneaeLiocranidae

Mu & Zhang, 2020

C11E2C92-B170-55F0-B71C-BCA2B507547A

Figs 1 , 2

Jacaena menglaensis Mu & Zhang, 2020: 343, figs 1H, 2C, 5D–F (♂).

##### Material examined.

1 ♂ (IZCAS-Ar 43812): **China**, Yunnan, Xishuangbanna, Mengla County, Menglun Town, XTBG, *Paramicheliabaillonii* plantation (about 20 years old), 21°54.772'N, 101°16.043'E, 608 m, collected by pitfall traps in leaf litter, 1–15 April 2007, G. Zheng leg; 1 ♂ (IZCAS-Ar 43813), same data as previous, but secondary tropical seasonal rain forest, 21°55.428'N, 101°16.441'E, 598 m, 9–13 August 2006; 1 ♂ (IZCAS-Ar 43814), same data as previous, but secondary tropical seasonal rain forest, 21°55.428'N, 101°16.441'E, 598 m, 16–31 July 2007; 1 ♀ (IZCAS-Ar 43815), same data as previous, but secondary tropical seasonal moist forest, 21°54.984'N, 101°16.982'E, 556 m, 1–9 September 2006; 1 ♀ (IZCAS-Ar 43816), same data as previous, but rubber plantation (about 20 years old), 21°54.684'N, 101°16.319'E, 585 m, 4–11 April 2007; 1 ♀ (IZCAS-Ar 43817), same data as previous, but secondary tropical seasonal moist forest, 21°54.718'N, 101°16.940'E, 645 m, 16–31 July 2007; 1 ♀ (IZCAS-Ar 43818), same data as previous, but secondary tropical seasonal moist forest, 21°54.607'N, 101°17.005'E, 633 m, 1–15 June 2007.

##### Diagnosis.

**Figure 1.**
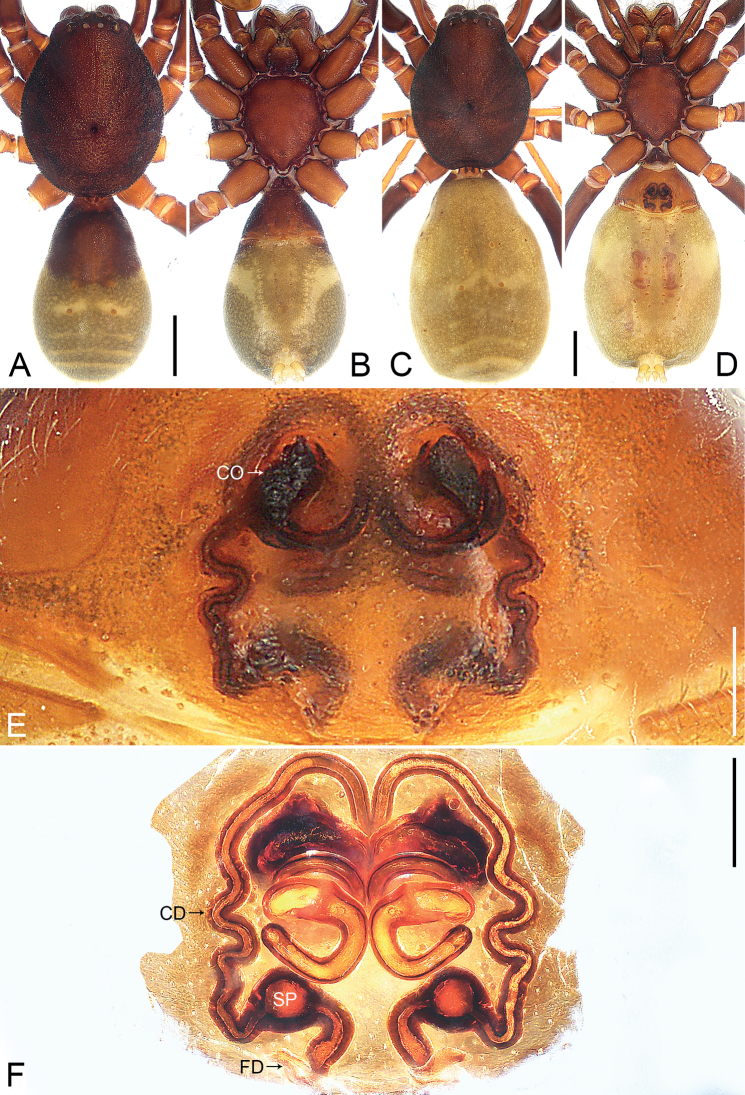
*Jacaenamenglaensis*, male (**A, B**) and female (**C–F**) **A** habitus, dorsal view **B** habitus, ventral view **C** habitus, dorsal view **D** habitus, ventral view **E** epigyne, ventral view **F** vulva, dorsal view. Abbreviations: CD = copulatory duct, CO = copulatory opening, FD = fertilization duct, SP = spermathecae. Scale bars: 1 mm (**A–D**); 0.2 mm (**E, F**).

**Figure 2.**
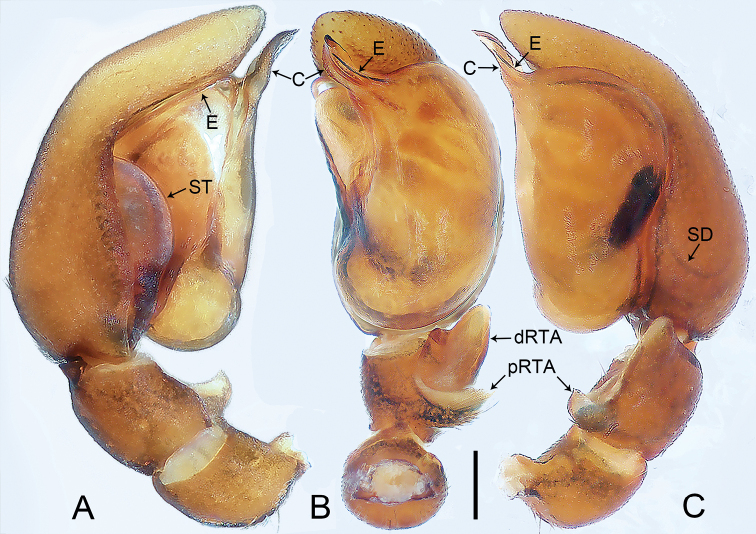
*Jacaenamenglaensis*, male **A–C** palp **A** prolateral view **B** ventral view **C** retrolateral view. Abbreviations: C = conductor, dRTA = distal process of retrolateral tibial apophysis, E = embolus, pRTA = proximal process of retrolateral tibial apophysis, SD = sperm duct, ST = subtegulum. Scale bar: 0.2 mm.

The species resembles *J.aspera* (cf. Figs 1, 2 with Mu and Zhang 2020: 336, figs 1d–f, 2d–f, 3a–e): the males have a similar embolus and retrolateral tibial apophysis (Fig. 2A–C), and the females have a similar spermatheca (Fig. 1F). Males can be distinguished by the membranous conductor (Fig. 2A–C; vs sclerotized conductor); females by the nearly elliptical copulatory openings (Fig. 1E; vs round copulatory openings), and by the laterally sinuous copulatory ducts (Fig. 1F; vs laterally vertical copulatory ducts). The species also resembles *J.zhui* (cf. Figs 1, 2 with Zhang and Fu 2011: 71, figs 1–15 and Mu and Zhang 2020: 345, figs 7a–i, 8a, b): the males have a similar embolus and retrolateral tibial apophysis (Fig. 2A–C), and the females have a similar fertilization duct (Fig. 1F). Males can be distinguished by the laminar conductor, which is slightly folded apically (Fig. 2A–C; vs apically helix-shaped conductor); females by the nearly elliptical copulatory openings (Fig. 1E; vs round copulatory openings), by the laterally sinuous copulatory ducts, which medially form three coils (Fig. 1F; vs copulatory ducts laterally vertical and medially forming two coils), and by the spermathecae situated in the posterior part of vulva (Fig. 1F; vs spermathecae larger, situated in the side of vulva).

##### Description.

**Male** (IZCAS-Ar 43812; Figs 1A, B, 2A–C): see Mu and Zhang (2020 figs 1h, 2c, 5d–f) for complete description.

**Female** (IZCAS-Ar 43815; Fig. 1C, D): total body length 8.13: carapace 3.43 long, 2.68 wide; opisthosoma 4.70 long, 3.07 wide. Carapace dark brown, oval, and strongly granulated, with faint radial grooves; fovea pit-like. Eye sizes and inter-distances: AME 0.13, ALE 0.15, PME 0.10, PLE 0.13; AME–AME 0.09, AME–ALE 0.07, PME–PME 0.15, PME–PLE 0.22, ALE–PLE 0.11; MOA 0.38 long, anterior width 0.31, posterior width 0.37. Chelicerae dark reddish brown, with three promarginal teeth, two retromarginal teeth. Endites and labium reddish brown, apically with a narrow membranous area; endites constricted in middle, converging apically. Labium 1.11 times longer than wide. Sternum and legs reddish brown. Leg spination as shown in Table 1. Leg measurements: I 9.06 (2.57, 0.99, 2.37, 2.02, 1.11); II 7.87 (2.30, 0.83, 1.98, 1.67, 1.09); III 6.77 (1.99, 0.72, 1.51, 1.52, 1.03); IV 10.10 (2.71, 1.03, 2.27, 2.66, 1.43). Opisthosoma ovoid, medially with four reddish apodemes, posterior half with four faint chevrons; venter reddish brown anteriorly and with two oblique pale stripes converging posteriorly. Spinnerets yellowish.

**Table 1.** Leg spination of *J.menglaensis*, female.

	I	II	III	IV
femur	3 pl			
tibia	7 pv and 7 rv	7 pv and 6 rv	2 pv and 1 rv	1 pl, 1 pv, 1 rl, 1 rv
metatarsus	4 pv and 4 rv	4 pl and 4 rl	1 pl, 1 pv, 1 rv	1 rl, 2 pv, 2 rv

***Epigyne*** (Fig. 1E, F). Epigynal plate length/width: 1.03/1.87, medially with two copulatory openings nearly elliptic. Vulva with copulatory ducts, laterally sinuous and tapered off, medially curved, forming three coils; spermathecae spherical, separated by more than their diameter from each other, situated in the posterior part of vulva; fertilization ducts pointing laterally.

##### Variations.

Males: total body length 6.15–6.84. Females: total body length 7.21–8.83.

##### Distribution.

China (Yunnan, type locality; Fig. 11).

#### ﻿Genus Oedignatha

Taxon classificationAnimaliaAraneaeLiocranidae

Thorell, 1881

C9A461BA-E809-51CE-824F-ED873C887B65

##### Type species.

*Oedignathascrobiculata* Thorell, 1881 from India.

##### Comments.

The genus includes 39 species from South and Southeast Asia. Only *O.platnicki* Song & Zhu, 1998 (♂♀) and *O.scrobiculata* (♂♀) are distributed in China.

#### ﻿ Oedignatha dian

Taxon classificationAnimaliaAraneaeLiocranidae

Lu & Li sp. nov.

70BACEBF-E3B2-539C-8219-DE7AEC75525E

https://zoobank.org/A9991F52-8CA6-4857-98E0-F2E94DBD0A7C

Figs 3 , 4 , 5 , 6

##### Type material.

***Holotype***: 1 ♂ (IZCAS-Ar 43819), **China**, Yunnan, Xishuangbanna, Mengla County, Menglun Town, XTBG, *Paramicheliabaillonii* plantation (about 20 years old), 21°54.772'N, 101°16.043'E, 608 m, collected by pitfall traps in leaf litter, 1–15 July 2007, G. Zheng leg. ***Paratypes***: 1 ♂ (IZCAS-Ar 43820), same data as holotype, but 21°53.823'N, 101°17.072'E, 613 m, 16–31 May 2007; 1 ♀ (IZCAS-Ar 43821), same data as holotype, but secondary tropical seasonal rain forest, 21°55.428'N, 101°16.441'E, 598 m, 1–9 October 2006; 1 ♀ (IZCAS-Ar 43822), same data as holotype, but rubber–tea plantation (about 20 years old.), 21°55.551'N, 101°16.923'E, 561 m, 16–31 July 2007.

##### Etymology.

The specific name refers to the type locality (Dian is a short name for Yunnan) and is a noun in apposition.

##### Diagnosis.

**Figure 3.**
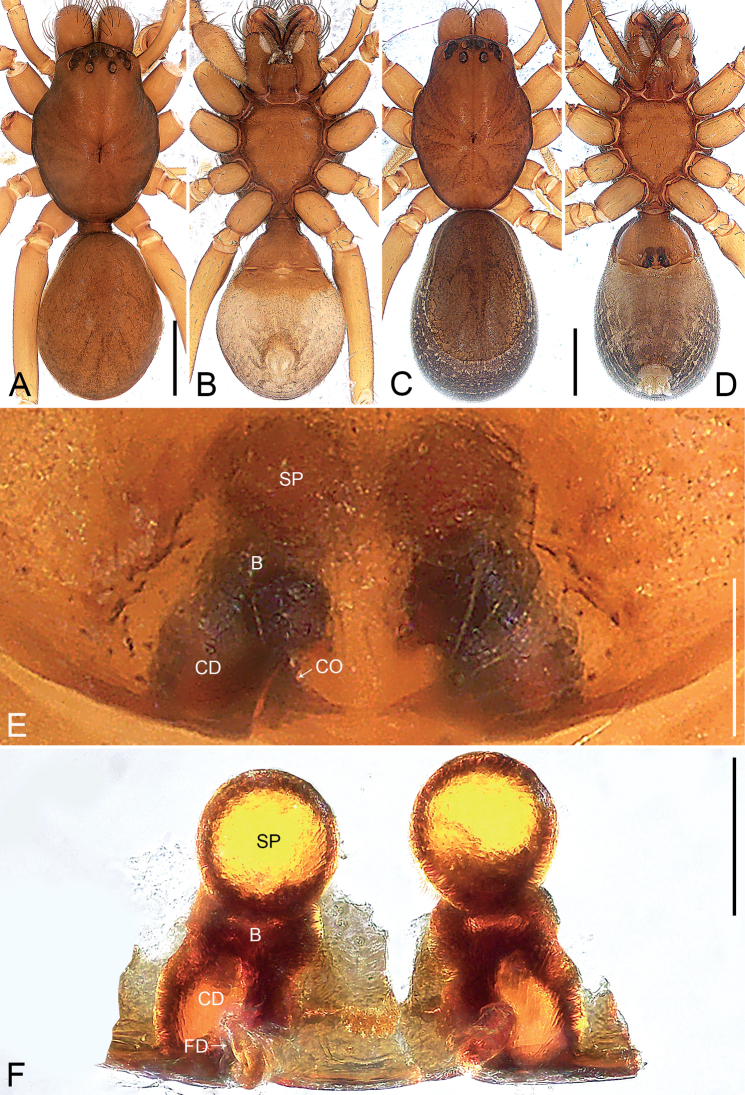
*Oedignathadian* sp. nov., holotype male (**A, B**) and paratype female (**C–F**) **A** habitus, dorsal view **B** habitus, ventral view **C** habitus, dorsal view **D** habitus, ventral view **E** epigyne, ventral view **F** vulva, dorsal view. Abbreviations: B = bursa, CD = copulatory duct, CO = copulatory opening, FD = fertilization duct, SP = spermathecae. Scale bars: 1 mm (**A–D**), 0.2 mm (**E, F**).

**Figure 4.**
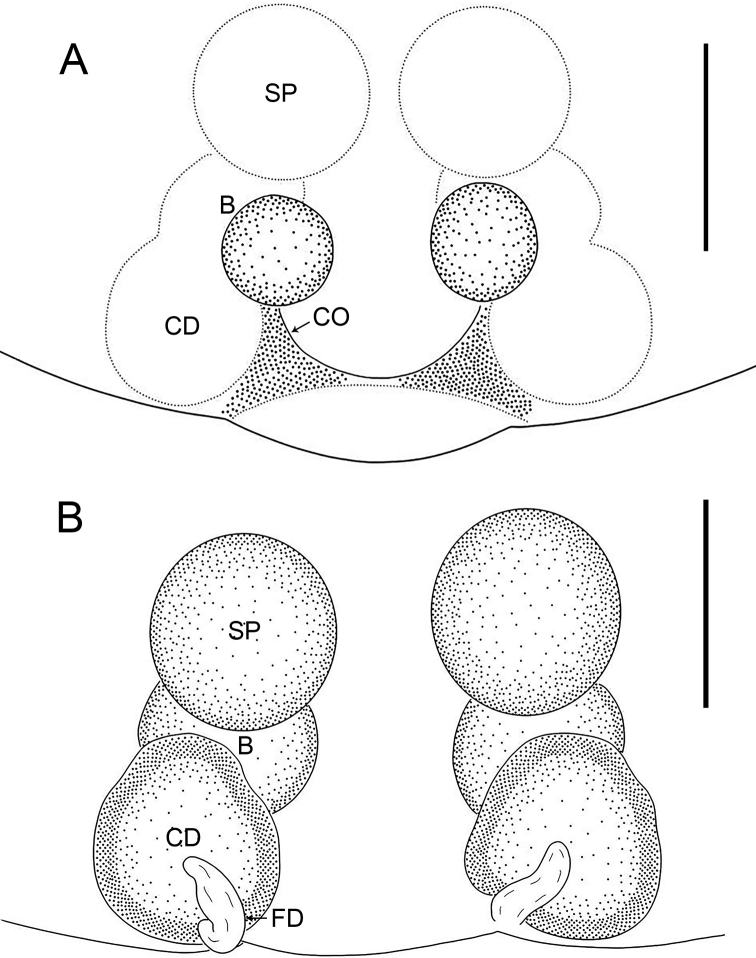
*Oedignathadian* sp. nov., paratype female (**A, B**) **A** epigyne, ventral view **B** vulva, dorsal view. Abbreviations: B = bursa, CD = copulatory duct, CO = copulatory opening, FD = fertilization duct, SP = spermathecae. Scale bars: 0.2 mm.

**Figure 5.**
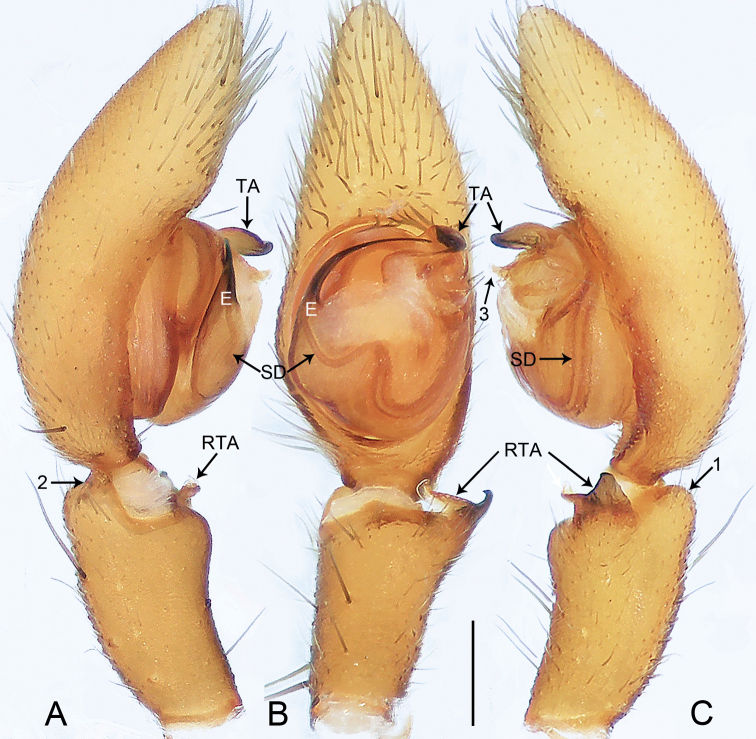
*Oedignathadian* sp. nov., holotype male **A–C** palp **A** prolateral view, arrow 2 points at prolateral process **B** ventral view **C** retrolateral view, arrow 1 points at dorsal hump, arrow 3 points at membranous outgrowth. Abbreviations: E = embolus, RTA = retrolateral tibial apophysis, SD = sperm duct, TA = tegular apophysis. Scale bar: 0.2 mm.

**Figure 6.**
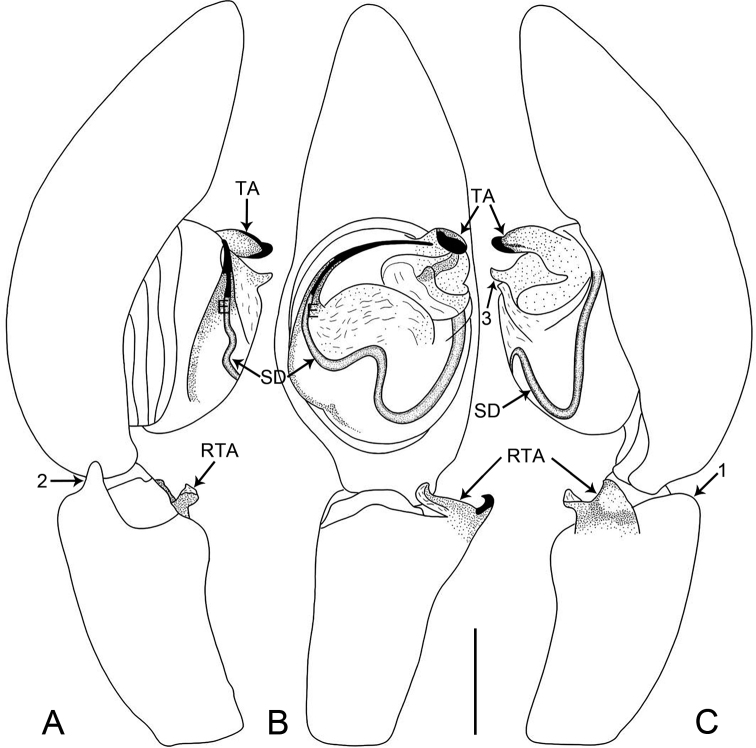
*Oedignathadian* sp. nov., holotype male **A–C** palp **A** prolateral view, arrow 2 points at prolateral process **B** ventral view **C** retrolateral view, arrow 1 points at dorsal hump, arrow 3 points at membranous outgrowth. Abbreviations: E = embolus, RTA = retrolateral tibial apophysis, SD = sperm duct, TA = tegular apophysis. Scale bar: 0.2 mm.

The new species resembles *O.barbata* Deeleman-Reinhold, 2001 (cf. Figs 3–6 and Deeleman-Reinhold 2001: 271, figs 362–366): the males have a similar retrolateral tibial apophysis (Figs 5B, C, 6B, C), and the females have similar bursae and spermathecae (Figs 3F, 4B). Males can be distinguished by the tegular apophysis entirely sclerotized distally and with small membranous outgrowth basally (arrow 3 in Figs 5C, 6C; vs tegular apophysis elliptical in ventral view, without these features), and by the tegulum with large membranous area in middle (Figs 5B, 6B; vs tegulum without membranous area); females by the epigyne with two round spots (Figs 3E, 4A; vs epigyne with two long elliptical strips).

##### Description.

**Male** (**holotype**, IZCAS-Ar 43819; Fig. 3A, B). Total body length 4.34: carapace 2.39 long, 1.67 wide; opisthosoma 1.95 long, 1.34 wide. Carapace reddish brown, sclerotized, with faint radial grooves and covered with pits, oval but strongly constricted at first coxae, lateral margins slightly sinuous; fovea as longitudinal slit-like. Eye sizes and interdistances: AME 0.12, ALE 0.11, PME 0.10, PLE 0.09; AME–AME 0.10, AME–ALE 0.08, PME–PME 0.23, PME–PLE 0.21, ALE–PLE 0.09; MOA 0.33 long, anterior width 0.34, posterior width 0.41. Clypeus with conical hump. Chelicerae reddish brown strongly protruding (length: 0.97) in anterior part and knee-shaped, and with basal protuberance, covered with long setae, bearing unique thin macrosetae medially crossing each other, with three promarginal teeth and five retromarginal teeth. Endites and labium reddish brown; endite constricted in middle, median margin grooved, subapically with large, semicircular membranous area, apical margin with long, curved setae. Labium 1.33 times longer than wide, with subbasal constriction. Sternum reddish brown. Legs yellowish. Leg spination as shown in Table 2. Leg measurements: I 9.31 (2.32, 0.62, 2.43, 2.39, 1.55); II 7.10 (1.93, 0.61, 1.71, 1.77, 1.08); III 5.86 (1.63, 0.44, 1.23, 1.59, 0.97); IV 8.19 (2.23, 0.55, 1.87, 2.35, 1.19). Opisthosoma reddish brown with faint reticulate pattern, oval, with large scutum covering entire dorsum surface; venter anteriorly reddish brown, posteriorly grey. Spinnerets grey.

**Table 2.** Leg spination of *O.dian* sp. nov., male.

	I	II	III	IV
femur	1 pl	1 d		
tibia	8 pv and 8 rv	7 pv and 6 rv	2 pl and 3 rv	2 pv and 3 rv
metatarsus	6 pv and 6 rv	5 pl and 4 rl	1 pv and 1 rv	1 pv and 1 rv

***Palp*** (Figs 5A–C, 6A–C). Tibia length/width: 0.54/0.25, distally with dorsal hump (arrow 1 in Figs 5C, 6C), prolateral triangular process (arrow 2 in Figs 5A, 6A), and with retrolateral tibial apophysis (length/width: 0.14/0.06) sclerotized, with dorsal branch the larger, triangular, and ventral branch thin, laminate. Cymbium long and narrow. Bulbus length/width: 0.42/0.37, 1/2 length of cymbium. Tegulum with distinct, sinuous sperm duct and large membranous area in middle. Tegular apophysis strongly sclerotized distally and with small membranous outgrowth basally (arrow 3 in Figs 5C, 6C). Embolus filiform, curved and tapered apically, originating from 8:00–9:30 o’clock on tegulum.

**Female** (**paratype**, IZCAS-Ar 43821; Fig. 3C, D). Total body length 5.46: carapace 2.56 long, 1.76 wide; opisthosoma 2.90 long, 1.82 wide. Color and somatic morphology as in male, except as noted. Eye sizes and interdistances: AME 0.13, ALE 0.12, PME 0.10, PLE 0.10; AME–AME 0.12, AME–ALE 0.09, PME–PME 0.21, PME–PLE 0.20, ALE–PLE 0.08; MOA 0.34 long, anterior width 0.37, posterior width 0.41. Leg measurements: I 9.76 (2.52, 0.66, 2.79, 2.57, 1.22); II 7.44 (2.24, 0.56, 1.94, 1.75, 0.95); III 6.35 (1.66, 0.58, 1.32, 1.75, 1.04); IV 9.12 (2.46, 0.64, 2.13, 2.56, 1.33). Opisthosoma brown, with scutum covering 4/5 of dorsum surface; venter of opisthosoma with epigastric scutum and with two brown stripes converging posteriorly; laterally with pale stripes.

***Epigyne*** (Figs 3E, F, 4A, B). Epigynal plate rectangular, length/width: 0.82/1.10, with two dark, round spots visible by transparency. Vulva with large spherical spermathecae, separated by less than their diameter from each other, roundish bursae and pair of fertilization ducts pointing antero-laterally. Copulatory openings wide, visible in ventral view. Copulatory ducts nearly elliptical in dorsal view, connecting bursae to spermathecae.

##### Variations.

Paratype male: total body length 5.21. Second paratype female: total body length 6.18.

##### Distribution.

China (Yunnan, type locality; Fig. 11).

##### Note.

The new species maybe identical to Oedignathacf.jocquei of Ramírez (2014: 233, fig. 161f–h, based on material from Ha Tinh, Vietnam). Further studies are necessary for solid conclusion.

#### ﻿ Oedignatha menglun

Taxon classificationAnimaliaAraneaeLiocranidae

Lu & Li sp. nov.

9A488A89-7576-567C-8F17-5A0B47FB4985

https://zoobank.org/9C5837D9-A2BD-40D2-B9E3-4462D099EEE6

Figs 7 , 8 , 9 , 10

##### Type material.

***Holotype***: 1 ♂ (IZCAS-Ar 43823), **China**, Yunnan, Xishuangbanna, Mengla County, Menglun Town, XTBG, Primary tropical seasonal rain forest, 21°57.445'N, 101°12.997'E, 744 m, hand catch in leaf litter, 4–11 May 2007, G. Zheng leg. ***Paratypes***: 1 ♂ (IZCAS-Ar 43824), same data as holotype, but 21°55.035'N, 101°16.500'E, 558 m, 19–26 May 2007; 1 ♀ (IZCAS-Ar 43825), same data as holotype, but secondary tropical seasonal moist forest, 21°54.984'N, 101°16.982'E, 656 m, collected by pitfall traps in leaf litter, 1–9 September 2006; 1♀ (IZCAS-Ar 43826), same data as holotype, but 21°55.035'N, 101°16.500'E, 558 m, 5–12 October 2006.

##### Etymology.

The specific name refers to the type locality and is a noun in apposition.

##### Diagnosis.

**Figure 7.**
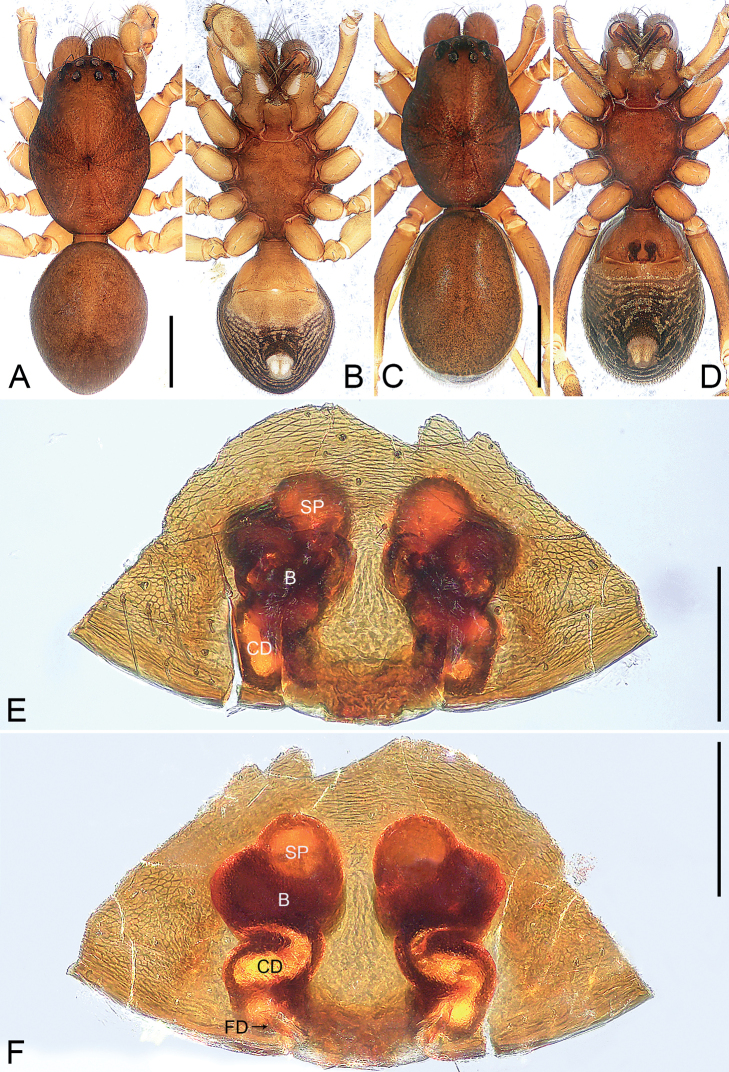
*Oedignathamenglun* sp. nov., holotype male (**A, B**) and paratype female (**C–F**) **A** habitus, dorsal view **B** habitus, ventral view **C** habitus, dorsal view **D** habitus, ventral view **E** epigyne, ventral view **F** vulva, dorsal view. Abbreviations: B = bursa, CD = copulatory duct, FD = fertilization duct, SP = spermathecae. Scale bars: 1 mm (**A–D**); 0.2 mm (**E, F**).

**Figure 8.**
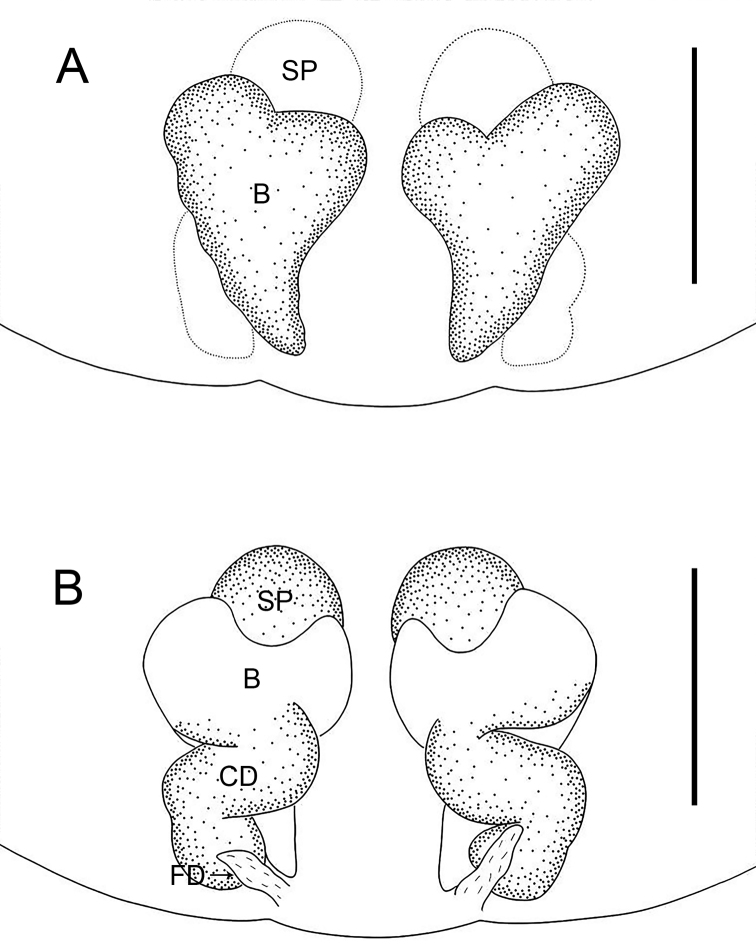
*Oedignathamenglun* sp. nov., paratype female (**A, B**) **A** epigyne, ventral view **B** vulva, dorsal view. Abbreviations: B = bursa, CD = copulatory duct, FD = fertilization duct, SP = spermathecae. Scale bars: 0.2 mm.

**Figure 9.**
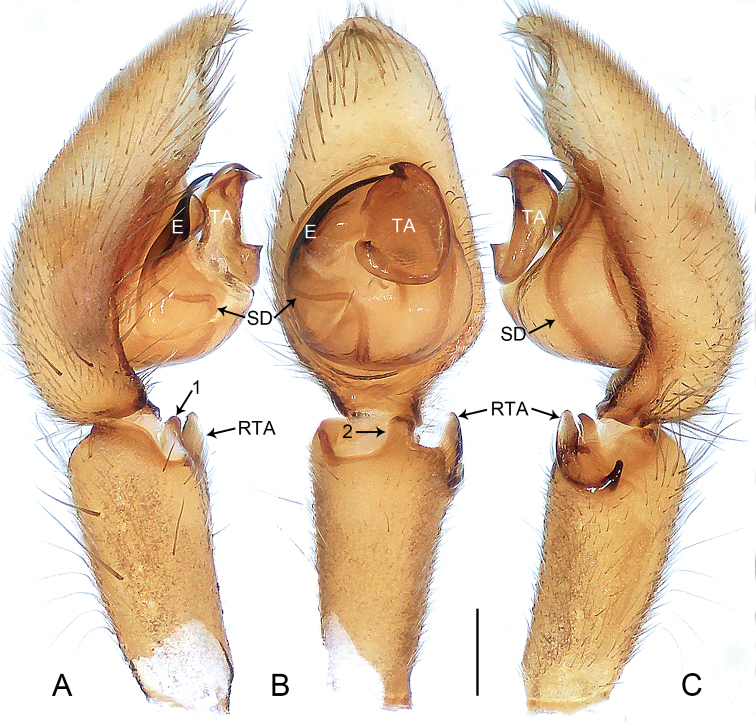
*Oedignathamenglun* sp. nov., holotype male **A–C** palp **A** prolateral view, arrow 1 points at ventral process **B** ventral view, arrow 2 points at ventral process **C** retrolateral view. Abbreviations: E = embolus, RTA = retrolateral tibial apophysis, SD = sperm duct, TA = tegular apophysis. Scale bar: 0.2 mm.

**Figure 10.**
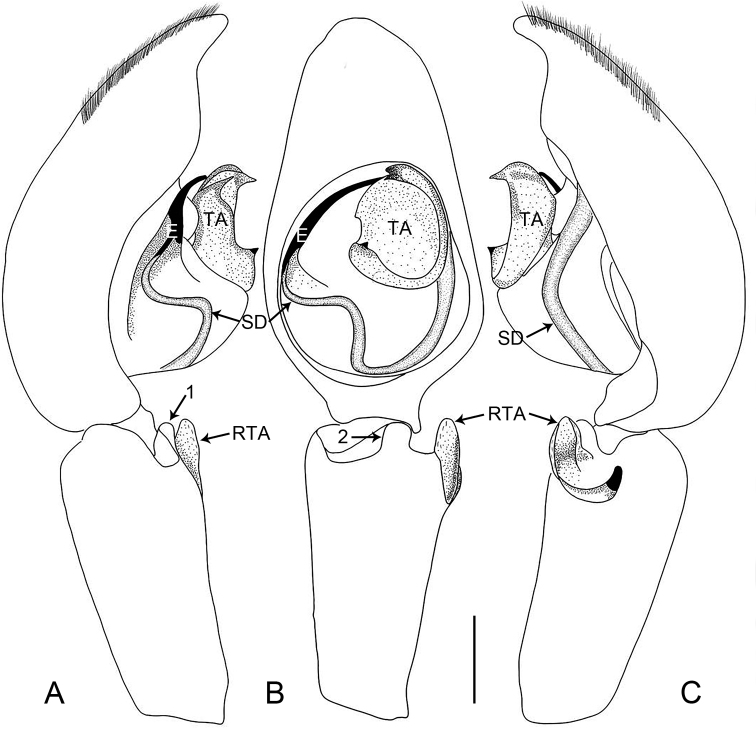
*Oedignathamenglun* sp. nov., holotype male **A–C** palp **A** prolateral view, arrow 1 points at ventral process **B** ventral view, arrow 2 points at ventral process **C** retrolateral view. Abbreviations: E = embolus, RTA = retrolateral tibial apophysis, SD = sperm duct, TA = tegular apophysis. Scale bar: 0.2 mm.

The new species resembles *O.jocquei* (cf. Figs 7–10 with Deeleman-Reinhold 2001: 268, figs 357–361): males have a similar embolus (Figs 9B, 10B) and females have a similar spermatheca (Figs 7F, 8B). Males can be distinguished by the tibia distally with a ventral rectangular process (arrow 1 in Figs 9A, 10A, arrow 2 in Figs 9B, 10B; vs tibia without ventral process), by the nearly round tegular apophysis with two sharp, sclerotized apophyses (Figs 9A–C, 10A–C; vs tegular apophysis bottle gourd-shaped, sclerotized apically), and by the retrolateral tibial apophysis curved in two parts: larger weakly sclerotized ventral apophysis with semitranslucent margin and thinner entirely sclerotized retrolateral apophysis (Figs 9A–C, 10A–C; vs two parts of retrolateral tibial apophysis entirely sclerotized); females can be distinguished by the reniform bursae (Figs 7E, 8A; vs spherical bursae), and by the tubular, curved copulatory ducts connecting bursae to the spermathecae (Figs 7F, 8B; vs vulva without obvious copulatory ducts).

##### Description.

**Male** (**holotype**, IZCAS-Ar 43823; Fig. 7A, B). Total body length 5.16: carapace 2.50 long, 1.71 wide; opisthosoma 2.66 long, 1.64 wide. Carapace reddish brown, sclerotized, with distinct radial grooves and covered with pits, oval but strongly constricted at first coxae, lateral margins slightly sinuous; fovea as longitudinal slit-like. Eye sizes and interdistances: AME 0.11, ALE 0.11, PME 0.09, PLE 0.09; AME–AME 0.11, AME–ALE 0.08, PME–PME 0.23, PME–PLE 0.19, ALE–PLE 0.07; MOA 0.32 long, anterior width 0.33, posterior width 0.41. Clypeus with conical hump. Chelicerae reddish brown strongly protruding (length: 0.98) in anterior part and knee-shaped, and with basal protuberance, covered with long setae, bearing unique thin macrosetae medially crossing each other, with three promarginal teeth, five retromarginal teeth. Endites and labium yellowish brown; endites constricted in middle, median margin grooved, subapically with large, semicircular membranous area, apical margin with long, curved setae. Labium 1.36 times longer than wide, with subbasal constriction. Sternum reddish brown. Legs yellowish. Leg spination as shown in Table 3. Leg measurements: I 7.12 (1.80, 0.65, 1.84, 1.80, 1.03); II 5.78 (1.60, 0.57, 1.33, 1.44, 0.84); III 5.19 (1.36, 0.59, 1.03, 1.36, 0.85); IV 6.93 (1.75, 0.60, 1.52, 1.98, 1.08). Opisthosoma reddish brown with faint reticulate pattern, oval, with large scutum covering the entire dorsum surface; venter yellowish, laterally with pale stripes. Spinnerets grey.

**Table 3.** Leg spination of *O.menglun* sp. nov., male.

	I	II	III	IV
femur	1 pl			
tibia	8 pv and 7 rv	7 pv and 5 rv	1 pl and 1 rv	2 pv and 3 rv
metatarsus	5 pv and 5 rv	5 pv and 4 rv	1 pl and 1 rl	1 pl and 1 rl

***Palp*** (Figs 9A–C, 10A–C). Tibia length/width: 0.71/0.40, distally with ventral rectangular process (arrow 1 in Figs 9A, 10A, arrow 2 in Figs 9B, 10B), and with retrolateral tibial apophysis (length/width: 0.17/0.20) curved in two parts: larger weakly sclerotized ventral apophysis with semitranslucent margin and thinner, entirely sclerotized, retrolateral apophysis. Cymbium nearly cylindrical. Bulbus length/width: 0.51/0.44, 1/2 length of cymbium. Tegulum with distinct, sinuous sperm duct. Tegular apophysis large, nearly round and with two sharp, sclerotized apophyses. Embolus filiform, slightly curved, larger at base, entirely sclerotized, originating from 8:00–9.30 o’clock on tegulum.

**Female** (**paratype**, IZCAS-Ar 43825; Fig. 7C, D). Total body length 4.03: carapace 2.08 long, 1.44 wide; opisthosoma 1.95 long, 1.16 wide. Color and somatic morphology as in male, except as noted. Eye sizes and interdistances: AME 0.10, ALE 0.09, PME 0.09, PLE 0.07; AME–AME 0.08, AME–ALE 0.08, PME–PME 0.17, PME–PLE 0.16, ALE–PLE 0.06; MOA 0.29 long, anterior width 0.29, posterior width 0.35. Endites and labium reddish brown. Legs brown. Leg measurements: I 6.21 (1.64, 0.60, 1.59, 1.56, 0.82); II 5.24 (1.46, 0.55, 1.24, 1.26, 0.73); III 4.56 (1.26, 0.52, 0.89, 1.19, 0.70); IV 6.48 (1.71, 0.57, 1.42, 1.80, 0.98). Venter of opisthosoma with reddish brown epigastric scutum, the posterior part dark brown with grey patterns. Spinnerets yellowish.

***Epigyne*** (Figs 7E, F, 8A, B). Epigyne simple, with rectangular plate, length/width: 0.61/1.01. Vulva with small spherical spermathecae, separated by less than their diameter from each other, reniform bursa and pair of fertilization ducts pointing antero-laterally. Copulatory openings not seen. Copulatory ducts tubular and curved, connecting bursae to spermathecae.

##### Variations.

Paratype male: total body length 4.70. Second paratype female: total body length 4.91.

##### Distribution.

**Figure 11.**
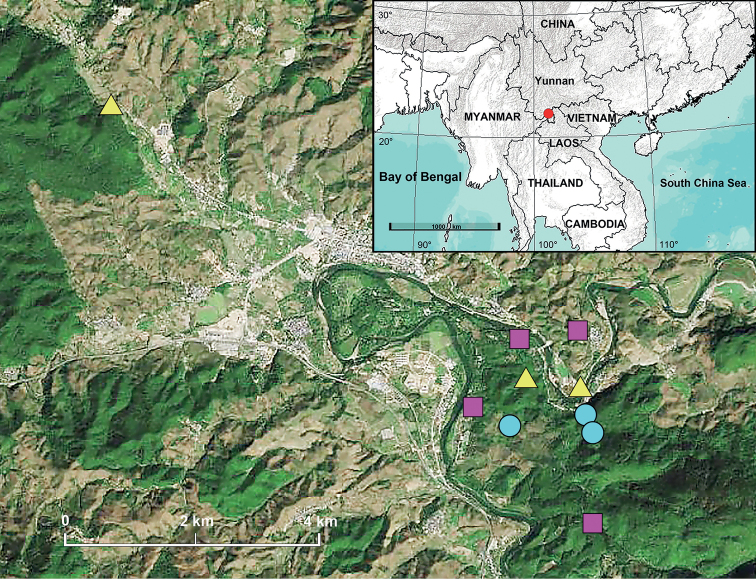
New distribution records of liocranid species from XTBG. Solid circle = *Jacaenamenglaensis*; square = *Oedignathadian* sp. nov.; triangle = *O.menglun* sp. nov.

China (Yunnan, type locality; Fig. 11).

## Supplementary Material

XML Treatment for Jacaena

XML Treatment for Jacaena menglaensis

XML Treatment for Oedignatha

XML Treatment for Oedignatha dian

XML Treatment for Oedignatha menglun
